# Hormones Matter? Association of the Menstrual Cycle With Selective Attention for Liked and Disliked Body Parts

**DOI:** 10.3389/fpsyg.2019.00851

**Published:** 2019-05-08

**Authors:** Kerstin Krohmer, Birgit Derntl, Jennifer Svaldi

**Affiliations:** ^1^Department of Psychology, University of Tübingen, Tübingen, Germany; ^2^Department of Psychiatry and Psychotherapy, University of Tübingen, Tübingen, Germany; ^3^Werner Reichardt Centre for Integrative Neuroscience (CIN), University of Tübingen, Tübingen, Germany; ^4^LEAD Graduate School and Research Network, University of Tübingen, Tübingen, Germany

**Keywords:** body image, body dissatisfaction, menstrual cycle, cycle phase, sexual hormones, attractiveness

## Abstract

Body dissatisfaction is wide spread among women and is considered a risk factor for eating pathology. Therefore, it is clinically relevant to investigate potential influencing factors. While previous research has mainly revealed inconsistent findings for attentional processes in body perception, the present study aimed to investigate the association of menstrual cycle phase with body satisfaction and its perception. In a within subject design, 16 women with a natural menstrual cycle (NC) and 19 women who used hormonal contraception (HC) rated their bodily attractiveness and underwent a 3-minute mirror exposure while their eye movements were recorded at two different times during their cycle (NC: ovulation vs. late luteal phase; HC: mid vs. end of cycle). At ovulation, NC women felt more attractive and gazed less at unattractive body parts in contrast to the late luteal phase, where they felt less attractive and gazed more at unattractive body parts. There was no difference in the gaze pattern for the attractive body parts at ovulation and late luteal phase. Notably, HC women showed a balanced gazed pattern at attractive and unattractive body at both times. The menstrual cycle phase is associated with women’s self-rated attractiveness and selective attention when looking at their own body. It should therefore be taken into account in clinical research addressing body image.

## Introduction

The undue influence of shape and weight on self-evaluation is a core feature of anorexia (AN) and bulimia nervosa (BN; American Psychiatric Association [APA], 2013), and also a clinical feature of individuals with binge eating disorder (BED; Grilo et al., 2010). Another aspect of body image is body dissatisfaction. Body dissatisfaction represents a negative evaluation of one’s own body including shape and weight (Garfinkel et al., 1992; Stice and Shaw, 2002). It is further considered a major risk factor for the development of eating pathology (Stice and Shaw, 2002; Stice et al., 2011) as it predicts higher levels of dieting and unhealthy weight control behavior (Neumark-Sztainer et al., 2006). Moreover, body dissatisfaction is a crucial factor for eating disorder maintenance (see Stice (2002) for a review) and relapse (Freeman et al., 1985; Keel et al., 2005; Masheb and Grilo, 2008). Therefore, it is clinically relevant to understand the underlying mechanisms of body dissatisfaction in order to develop more effective prevention programs.

Although body dissatisfaction is not exclusively a problem in women, it has been shown that women report higher levels of body dissatisfaction than men (Borchert and Heinberg, 1996; Cash et al., 2004; Purton et al., 2019) and furthermore a significant proportion of women are dissatisfied with their own body (Frederick et al., 2006). Taking into consideration that women have a higher risk for developing an eating disorder, especially AN and BN (Smink et al., 2012), it is important to set the focus on this group in body dissatisfaction research.

Cognitive theories propose that body dissatisfaction stems from activation of the negative cognitive and affective components of the body schema (Vitousek and Hollon, 1990; Williamson et al., 2004). Accordingly, this body schema is activated by confrontation with body-related information (e.g., a mirror or internal reminders of body-related situations) and, as a consequence, directs individuals’ attention to (but also memory and interpretation of) body-related cues. At the behavioral level, such distortions in the information processing stream are observable as body checking or avoidance of certain body parts, but also pathological eating behavior (Williamson et al., 2004).

Empirical evidence conducted both in the subclinical (for a review see Rodgers and DuBois, 2016) as well as the clinical domain (for reviews see Lewer et al., 2017; Jiang and Vartanian, 2018) indeed supports the presence of attentional biases when processing self and other bodies as well as body parts, albeit with some inconsistencies. In non-clinical samples, higher levels of body image concerns and drive for thinness were associated with increased attention toward unattractive body parts of one’s own body (Jansen et al., 2005; Roefs et al., 2008) and others’ body parts that are related to weight gain (Hewig et al., 2008). Furthermore, women with high levels of body dissatisfaction showed increased attention toward thin and fat bodies (Gao et al., 2013, 2014). By contrast, other studies conducted with women with high levels of body dissatisfaction or drive for thinness yielded evidence of attentional avoidance of other bodies (Lykins et al., 2014), their own body (Janelle et al., 2009) and especially of critical body parts that are indicative of a person’s weight (Janelle et al., 2003).

Similar conflicting findings have also been reported for the clinical domain. In AN, one study indicated an attentional bias toward images of thin and fat bodies (Pinhas et al., 2014), while others reported a hypervigilance for body parts (of others) often indexed as unattractive (Horndasch et al., 2012). Further, in studies that assessed the processing of one’s own body, women with AN (compared to controls) were characterized by a shorter gaze duration (von Wietersheim et al., 2012), but also longer and more frequent gazes toward negatively valenced body parts (Freeman et al., 1991; Tuschen-Caffier et al., 2015; Bauer et al., 2017).

While the considerable methodological variability in the measurement of attentional processes certainly makes a comparison of the respective studies challenging, research so far has mostly neglected possible state factors implicated in body-related information processing. Indeed, cognitive models emphasize that state factors, especially those that activate weight concerns (or reduce these), might cause body image to worsen (or improve) (Williamson et al., 2004). While some studies observed significant effects of mood on the attentional processing of one’s own body (Tuschen-Caffier et al., 2015; Svaldi et al., 2016), a completely neglected factor in this context has been the possible influence of the female menstrual cycle and thus sex hormone fluctuations, even though evidence suggests that perceived attractiveness and body dissatisfaction is highly influenced by the menstrual cycle (e.g., Carr-Nangle et al., 1994; Haselton and Gangestad, 2006; Durante et al., 2008).

Simply put, the menstrual cycle is separated into the follicular and the luteal phase. The follicular phase includes the first half of the cycle, from menstruation to the day prior to ovulation. The luteal phase refers to the second half of the cycle, from ovulation to the last day before the next menstruation (Yen, 1979; Asso, 1984; Silverthorn et al., 2004). During the menstrual cycle, women are exposed to large fluctuations in sex hormones (Sherman and Korenman, 1975). While the follicular phase is characterized by a low concentration of progesterone and a slow rise of the estradiol level with its peak before ovulation, the luteal phase consists of a rise in progesterone and a moderately high estradiol level. The luteal phase initiates with ovulation, which marks the most fertile time (about 2 to 4 days) of the female menstrual cycle. At the hormonal level, it is initiated by a quick rise of the luteinizing hormone (LH) (Sundström Poromaa and Gingnell, 2014). These hormonal fluctuations are related to different physical changes, such as water retention, increased appetite, and changed autonomic reactivity (Moos et al., 1969; Bancroft and Bäckström, 1985). In addition to these physical reactions, a number of psychological changes occur, including increased negative affect and mood as well as augmented reward sensitivity (for a review see Sundström Poromaa and Gingnell, 2014).

Related to the concept of body dissatisfaction, women were shown to feel more physically attractive in the follicular phase, close to the time of ovulation, than in the luteal phase (Haselton and Gangestad, 2006; Durante et al., 2008). Additionally, female faces have been judged more attractive on photographs that were taken during ovulation than photographs taken during the luteal phase (Roberts et al., 2004).

Taken together, first evidence suggests that both self and other perceived attractiveness is affected by the menstrual cycle and is highest during the most fertile phase, i.e., ovulation. With regard to body dissatisfaction, studies reported reduced dissatisfaction with one’s own body during the follicular relative to the luteal phase (Carr-Nangle et al., 1994; Racine et al., 2012). Supposedly, this difference is dependent on the progesterone level, which is low in the follicular and high in the luteal phase (Racine et al., 2012).

In summary, fluctuations in sex hormones during the menstrual cycle have been shown to be implicated in self-reported perceived attractiveness and body dissatisfaction. On this background, conflicting findings reported by previous studies on attentional biases may be partly explained by the lack of controlling for the impact of the menstrual cycle.

Therefore, the aim of the present study was to test whether the menstrual cycle is associated with a dysfunctional attention allocation toward the self-defined most attractive and unattractive region of one’s own body. To this end, eye movements were recorded twice during a short mirror exposure task in naturally cycling (NC) women and women using hormonal contraceptives. One session was held during the high fertility days in the middle of the cycle where ovulation takes place and progesterone is low. The other session was conducted during the low fertility days in the late luteal phase when progesterone is high. Women who used HC (oral contraception or vaginal contraceptive ring) served as control group, as for them estradiol and progesterone levels across the cycle are relatively constant (Rivera et al., 1999).

We expected that NC women would feel less attractive during the late luteal phase and would show longer and more frequent gazes toward their most unattractive body part relative to the most attractive. In contrast, during ovulation NC women were expected to feel more attractive and show shorter and less frequent gazes toward their most unattractive body part and more toward their most attractive. For the HC taking women we expected no gaze difference between the two testing sessions due to the restricted fluctuation of sex hormones.

## Materials and Methods

### Participants

A group of 37 female students of the local University participated in the study. Participants were recruited by means of University announcements and Email. Two participants had to be excluded because of technical problems during the lab session. Thus, a sample of 35 women remained. Sixteen of them were naturally cycling and had an ovulatory cycle (NC Group) whereas 19 of them used combined oral contraceptives or a vaginal contraceptive ring [HC (hormonal contraceptives) Group; 16 taking oral contraceptives].

The inclusion criterion for the NC Group was no HC for at least 6 months and for the HC Group HC for at least 6 months. Exclusion criteria for both groups included a current or lifetime diagnosis of an eating disorder (self-report), a body mass index (BMI) outside the normal weight range according to the World Health Organization (WHO), defined as <18.5 and >24.9 (WHO, 2000) and a current or past pregnancy (Clark et al., 2009).

The mean (*M*) cycle length of the women in the NC Group was 28.43 [standard deviation (*SD*) = 3.96] days and mean time of HC in the HC Group was 43.33 (*SD* = 23.69) months. The mean age of the sample was 20.77 (*SD* = 2.12) years (range: 18 to 27 years). Both groups did not differ in age, BMI, depression scores and body dissatisfaction, as measured by the Beck Depression Inventory (BDI; Beck et al., 1996) and the Body Shape Questionnaire (BSQ; Cooper et al., 1987), respectively. There was, however, a significant group difference for relationship status, with the HC Group containing more participants in a committed relationship than in the NC Group (see Table 1 M, SD and statistics).

**Table 1:** Sociodemographic information of women in the NC Group and the HC Group.

	NC Group *N* = 16 *M* (*SD*)	HC Group *N* = 19 *M* (*SD*)	* Test statistics*	* p*
Mean age (years)	20.69 (2.39)	20.84 (1.92)	*t* (33) = –0.21	0.833
BMI (kg/m^2^)^1^	20.98 (2.14)	21.47 (1.78)	*t* (25) = –0.63	*0.537*
BDI-II	5.69 (6.22)	4.68 (5.05)	*t* (33) = 0.53	*0.601*
BSQ	56.50 (15.28)	54.26 (12.00)	*t* (33) = 0.49	*0.631*

	*** N* (%)**	***N* (%)**	**χ^2^**	***p***

Relationship status				
single	11 (68.75)	6 (31.58)	χ2 (1) = 4.80	0.**044**
mated	5 (31.25)	13 (68.42)		


*BMI, Body Mass Index; BDI-II, Becks Depression Inventory; BSQ, Body Shape Questionnaire. ^*1*^ Eight participants did not provide details about height and weight.*

The study was approved by the Ethics Committee of the Medical Faculty of the University and the University Hospital of Tübingen and written informed consent was obtained from all participants. Participants were remunerated either by course credits or with ten euro.

### Questionnaires

The following self-report measurements were administered: (1) The BSQ; Cooper et al., 1987; German Version: Waadt et al., 2013) is a reliable and valid measure assessing body dissatisfaction, with high scores for internal consistency and split-half reliability > 0.90 (Pook et al., 2002). Each item [e.g., “Have you been afraid that you might become fat (or fatter)?”] is scored 1 to 6 with “never” = 1 and “always” = 6. The overall score is the sum of all 34 item scores and higher scores represent higher body dissatisfaction. Internal consistency in the present sample was high with Cronbach’s alpha (α) = 0.94. (2) The Beck Depression Inventory (BDI-II) was used to assess severity of depression (Beck et al., 1996; Kühner et al., 2007). This questionnaire is a widely used instrument to assess the severity of depression with high scores for internal consistency (α > 0.80) and retest reliability (*rtt* > 0.60). It contains 21 items, with each item scored on a scale value of 0 (e.g., “I do not feel sad”) to 3 (e.g., “I am so sad or unhappy that I can’t stand it”). Internal consistency for the present sample was high with α = 0.87. (3) The Positive and Negative Affect Scale (PANAS, Watson et al., 1988; Krohne et al., 1996) was used to assess participants’ mood prior to the mirror task. It is a reliable and valid measure to assess positive and negative affect with high scores for internal consistency (α > 0.80). The scale consists of 20 adjectives (10 positive, e.g., “inspired” and 10 negative, e.g., “upset”) being rated from “very slightly or not at all” = 1 to “extremely” = 5. Separate subscales were calculated for positive and negative affect. Internal consistency for the present sample was borderline acceptable for the two subscales with α > 0.68. (4) Two 10 cm visual analog scales (VAS) anchored “not at all attractive” and “extremely attractive” were used for the assessment of momentary self-perceived attractiveness (Durante et al., 2008). The items were “At the moment I feel…“ and “At the moment potential partners find me….” Participants filled in these items prior to each mirror task. (5) A ranking for participants’ own body parts (shoulders, arms, hands, décolleté, breast, stomach, hips, and thighs) from most unattractive ( = 1) to most attractive ( = 8), was used to identify the most attractive and the most unattractive body part for each participant.

### Procedure

Interested women were prescreened via telephone and invited for participation if none of the exclusion criteria were reported. Every participant was scheduled to come to the lab for two sessions – one on an expected high fertility day at mid cycle and one on an expected low fertility day at the cycle end. Prior to the lab sessions, order of testing (mid cycle vs. cycle end) was counterbalanced across groups.

#### Assessment of Menstrual Cycle Phase in NC Women

To ensure accuracy of the menstrual phase, NC women were instructed to contact the investigator on the first day of their menses following the first phone contact, and contact her again on the first day of their next menses in order to determine the length of their menstrual cycle. Thus, one cycle was monitored before inclusion in the study. Based on this information, participants were handed six LH test strips (One Step^®^ Ovulation Tests at 20 mlU/mL) 4 days before the expected ovulation to determine the exact day of ovulation. Participants used the tests at home and were instructed to call when a LH surge was detected (positive LH test). The lab session took place within the following 48 h. The session during the late luteal phase (cycle end) took place 10–12 days after ovulation, i.e., 4 to 2 days prior to their next menses.

#### Assessment in the HC Group

As HC impedes ovulation (Rivera et al., 1999), lab sessions for HC women were scheduled at the 14th to 16th day of the cycle (similar to ovulation at mid cycle) and the 24th to 26th day (similar to late luteal phase at cycle end) in counterbalanced order.

The procedure of the two lab sessions was identical for both groups. At the beginning of each session, participants filled in the PANAS and the VAS to assess current mood and current self-perceived attractiveness, respectively. They then changed into standardized underwear (beige top and panties) and were instructed to stand in front of a closed mirror (distance 1.2 m) wearing the eye tracking device (following the procedure utilized by Tuschen-Caffier et al., 2015). SensoMotoric Instruments’ (SMI) wearable eye tracking glasses were used, which are similar in appearance to regular glasses, with a scene camera that captures the scene in front of the participant and two infrared cameras looking at the participant’s eyes and estimating their gaze location.

Following calibration, the mirror was uncovered, eye movement recording was started and participants were instructed to look at their body for 3 min in the absence of the investigator. After 3 min, the investigator came back, covered the mirror, stopped the recording and removed the eye tracking glasses.

Following the second session, participants completed the ranking of their body parts. Between the two lab sessions participants were asked to fill in a sociodemographic questionnaire, the BSQ and the BDI-II via the online survey tool Unipark (by Questback©).

### Data Preprocessing

The recorded eye tracking data was preprocessed with the SMI analysis program BeGaze. A region analysis was used. Every fixation was mapped on a reference view consisting of a picture of a female silhouette. After that, areas of interest (AOIs) for shoulder, arms, hands, décolleté, breast, stomach, hips, and thighs were defined and fixation durations and fixation frequency for the AOIs were calculated and set into relation to the total fixation duration and frequency within the 3-minute measure. For each participant only the body parts defined as the most attractive and the most unattractive were considered for the analysis. The dependent variable here was therefore the proportion of fixation duration and frequency for the most attractive and unattractive body part of the total fixation time and frequency (during the 3 min measurement).

### Statistical Analyses

Data analysis was carried out using the Statistical Package for the Social Sciences (SPSS, Version 25). Differences between the groups in sociodemographic variables (e.g., age, BMI, etc.) were analyzed using two-sample *t*-tests for independent samples. For the categorical data, Chi –Square tests were used. In order to ensure that the two groups did not differ in mood prior to the mirror task a Cycle (mid cycle vs. cycle end) × Group (NC Group vs. HC Group) mixed analysis of variance (ANOVA) for the positive and negative scale (PANAS) was calculated. For the attractiveness ratings, again a mixed ANOVA with Cycle and Group as factors was conducted.

The gaze data was analyzed using a mixed ANOVA with the within-subject factors Body Region (most attractive vs. most unattractive) and Cycle (mid cycle vs. cycle end) and the between-subjects factor Group (NC Group vs. HC Group). To disentangle significant interaction effects, subsequent ANOVAs and groupwise paired *t*-tests were performed. Given directional hypotheses (Kimmel, 1957; Ruxton and Neuhäuser, 2010), one-tailed *t*-tests were applied. In all of the analyses, the significance level was *p* = 0.05. For significant results, effect sizes were calculated (partial eta squared or Cohen’s *d*). The assumption of normality was satisfied in both groups for the gaze data (Kolmogoroff–Smirnoff *p*s > 0.07) and the attractiveness VAS rating (Kolmogoroff–Smirnoff *p*s > 0.80).

## Results

### Mood Assessment Prior to Mirror Exposure

Groups did not differ in positive and negative affect prior to the mirror exposure at the two time points (Interaction Group × Cycle: Positive affect: *F* (1,33) = 2.19, *p* = 0.149, $\eta_{P}^{2}$ = 0.03; Negative affect: *F* (1,33) = 0.09, *p* = 0.386, $\eta_{P}^{2}$ < 0.01). See Table 2 for means (*M*) and standard deviations (*SD*).

**Table 2:** Means (standard deviation) for the positive and negative affect, measured with the PANAS prior to the mirror task.

		NC Group *M* (*SD*)	HC Group *M* (*SD*)
Positive Affect	high fertility	30.44 (6.55)	30.32 (6.64)
	low fertility	27.25 (5.22)	29.79 (5.78)
Negative Affect	high fertility	12.94 (2.54)	12.11 (2.18)
	low fertility	13.13 (2.68)	12.11 (1.88)


*PANAS, Positive and Negative Affect Scale.*

### Attractiveness Ratings

The 2 (Group) × 2 (Cycle) mixed ANOVA for the self-rated attractiveness yielded no significant main effect of Cycle [*F* (1,33) = 0.28, *p* = 0.300, $\eta_{P}^{2}$ < 0.01], but a significant main effect of Group [*F* (1,33) = 3.48, *p* = 0.036, $\eta_{P}^{2}$ = 0.10] and a significant interaction of Group × Cycle, *F* (1,33) = 3.02, *p* = 0.046, $\eta_{P}^{2}$ = 0.08. As depicted in Table 3, self-rated attractiveness from mid to end of cycle decreased in the NC women and increased in the HC women, however, these changes were not significant in either group [NC women: *t* (15) = 1.26, *p* = 0.113, *d* = 0.42; HC women: *t* (18) = -1.16, *p* = 0.131, *d* = -0.24]. The difference in the attractiveness rating between the groups was not significant at mid cycle, *t* (33) = -0.56, *p* = 0.288, *d* = -0.28, but at cycle end the HC Group reported higher self-rated attractiveness than the NC Group, *t* (33) = -2.67, *p* = 0.006, *d* = -0.91.

**Table 3:** Means (standard deviation) for the attractiveness VAS ratings for mid cycle and cycle end in dependence of group membership.

		NC Group *M* (*SD*)	HC Group *M* (*SD*)
VAS Item: “At this moment I feel	high fertility	5.43 (1.84)	5.76 (1.70)
… attractive”
	low fertility	4.76 (1.32)	6.12 (1.46)
VAS Item: “At this moment	high fertility	5.84 (1.61)	6.52 (1.81)
potential partners find me…”
	low fertility	4.79 (1.44)	6.96 (1.50)


*VAS, Visual analog scale.*

The mixed ANOVA on the attractiveness rating regarding potential partners revealed no significant main effect of Cycle [*F*(1,33) = 0.78, *p* = 0.192, $\eta_{P}^{2}$ = 0.01], but a significant main effect of Group [*F* (1,33) = 11.16, *p* < 0.001, $\eta_{P}^{2}$ = 0.26], with higher ratings in HC women, and a significant interaction of Group × Cycle, *F*(1,33) = 4.60, *p* = 0.020, $\eta_{P}^{2}$ = 0.12. Group-wise paired *t*-tests disentangling the significant interaction showed that NC women rated their attractiveness for potential partners higher at mid cycle than at the cycle end, *t*(15) = 2.03, *p* = 0.030, *d* = 0.69. Notably, ratings did not differ in terms of time in HC women, *t*(18) = -0.94, *p* = 0.180, *d* = -0.26. Again the difference in the attractiveness rating between the groups was not significant at mid cycle, *t*(33) = -1.17, *p* = 0.126, *d* = -0.58, but at cycle end the HC Group reported higher self-rated attractiveness than the NC Group, *t*(33) = -3.83, *p* < 0.001, *d* = -1.31. See Table 3 for means (*M*) and standard deviations (*SD*).

### Gaze Data – Duration

**FIGURE 1:**
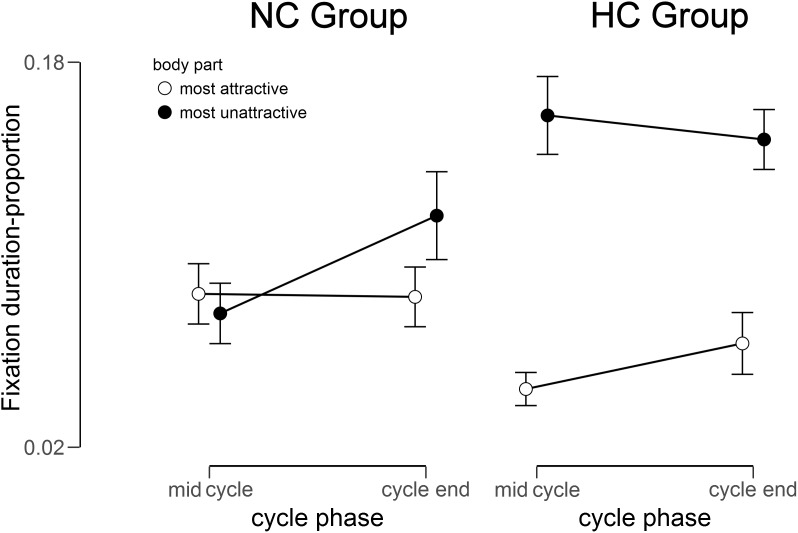
Fixation duration for the most unattractive and most attractive body part in relation to the total fixation duration within the mirror exposure for the NC (natural menstrual cycle) Group and the HC (hormonal contraceptive) Group at mid cycle and cycle end.

The 2 (Group) × 2 (Body Region) × 2 (Cycle) mixed ANOVA for gaze duration showed no significant main effects of Group (*F* (1, 33) = 0.94, *p* = 0.170, $\eta_{P}^{2}$ = 0.03) and Cycle [*F*(1,33) = 1.79, *p* = 0.095, $\eta_{P}^{2}$ = 0.05], but a significant main effect of Body Region [*F* (1,33) = 22.58, *p* < 0.001, $\eta_{P}^{2}$ = 0.41], as all participants gazed longer toward their most unattractive in relation to their most attractive body part. Moreover, a significant interaction of Group × Body Region [*F* (1,33) = 13.51, *p* < 0.001, $\eta_{P}^{2}$ = 0.29], and a significant 3-way interaction of Group × Body Region × Cycle, *F* (1,33) = 5.40, *p* = 0.013, $\eta_{P}^{2}$ = 0.14, emerged (see Figure 1). There were no other significant interactions (all *F*s < 1.80, all *p*s > 0.100).

To disentangle the 3-way interaction, a groupwise 2 (Cycle) × 2 (Body Region) ANOVA was conducted. For NC women this yielded no significant main effects (all *F*s < 2.30, all *p*s > 0.100, all $\eta_{P}^{2}$ < 0.130), but a significant interaction of Body Region × Cycle, *F* (1,15) = 3.66, *p* = 0.038, $\eta_{P}^{2}$ = 0.20, whereby participants spent more time looking at the most unattractive relative to the most attractive part at the cycle end [*t*(15) = -1.92, *p* = 0.037, *d* = 0.63], while there was no such difference at mid cycle [*t*(15) = 0.12, *p* = 0.453, *d* = 0.02).

For HC women analysis yielded a significant main effect of Body Region [*F*(1,18) = 43.43, *p* < 0.001, $\eta_{P}^{2}$ = 0.71], whereby participants spent more time looking at the most unattractive than at the most attractive part, *t*(18) = -6.59, *p* < 0.001, *d* = 1.86. There was, however, no significant interaction [*F*(1,18) = 1.91, *p* = 0.092, $\eta_{P}^{2}$ = 0.10] or main effect of Cycle [*F*(1,18) = 0.14, *p* = 0.358, $\eta_{P}^{2}$ = 0.01].

### Gaze Data – Frequency

**FIGURE 2:**
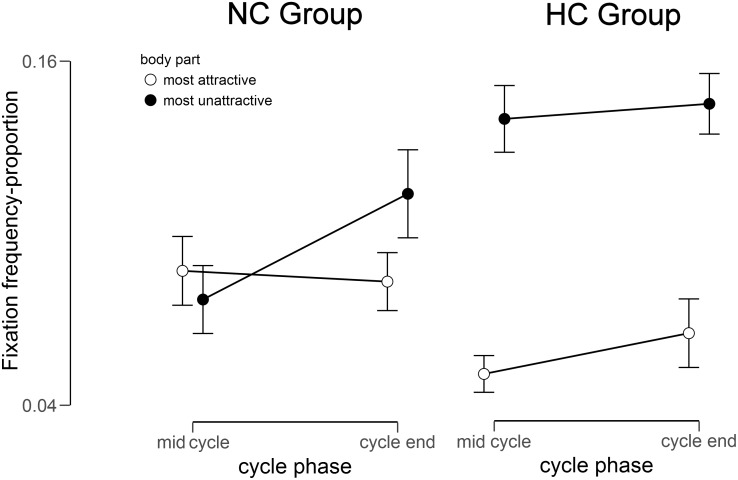
Fixation frequency for the most unattractive and most attractive body part in relation to the total fixation frequency within the mirror exposure for the NC (natural menstrual cycle) Group and the HC (hormonal contraceptive) Group at mid cycle and cycle end.

The 2 (Group) × 2 (Body Region) × 2 (Cycle) mixed ANOVA for the gaze frequency resulted in a significant main effect of Body Region [*F*(1,33) = 17.25, *p* < 0.001, $\eta_{P}^{2}$ = 0.34] and Cycle [*F*(1,33) = 4.18, *p* = 0.025, $\eta_{P}^{2}$ = 0.11], but no main effect of Group [*F*(1,33) = 0.56, *p* = 0.230, $\eta_{P}^{2}$ = 0.02]. Moreover, a significant 2-way interaction of Group × Body Region [*F*(1,33) = 10.49, *p* = 0.002, $\eta_{P}^{2}$ = 0.24] and a significant 3-way interaction of Group × Body Region × Cycle, *F* (1,33) = 5.83, *p* = 0.011, $\eta_{P}^{2}$ = 0.15, emerged (see Figure 2). No further interaction reached significance (all *p*s > 0.082).

To disentangle the 3-way interaction, additional 2 (Cycle) × 2 (Body Region) ANOVAs were performed. For NC women no significant main effects of Body Region [*F*(1,15) = 2.56, *p* = 0.065, $\eta_{P}^{2}$ = 0.15] and Cycle [*F*(1,15) = 0.34, *p* = 0.285, $\eta_{P}^{2}$ = 0.02] occurred, but a significant interaction of Body Region × Cycle, *F*(1,15) = 7.44, *p* = 0.008, $\eta_{P}^{2}$ = 0.33. Subsequent paired *t*-tests showed that gaze frequency toward the most unattractive relative to the most attractive body part was higher at the cycle end, *t*(15) = -2.27, *p* = 0.020, *d* = 0.73, while there was no such difference at mid cycle, *t*(15) = 0.43, *p* = 0.337, *d* = 0.05. By contrast for HC women, an ANOVA yielded a significant main effect for Body Region, *F*(1,18) = 33.54, *p* < 0.001, $\eta_{P}^{2}$ = 0.65, whereby participants’ gaze frequency was overall higher for the most unattractive relative to the most attractive part. There were no other significant main effects or interactions (all *F*s < 2.60, all *p*s > 0.100, all $\eta_{P}^{2}$ < 0.150).

## Discussion

The aim of the present study was to test the association of menstrual cycle phase with the attentional processing of one’s own body in a sample of healthy female students. Based on previous results which highlight the impact of the menstrual cycle on self-perceived attractiveness (Carr-Nangle et al., 1994; Haselton and Gangestad, 2006; Durante et al., 2008; Racine et al., 2012), it was hypothesized that naturally cycling women would show longer and more frequent gazes toward their most unattractive relative to the most attractive body part during the late luteal phase, i.e., last days of the menstrual cycle. A reversed pattern was expected for ovulation (mid cycle). Further, we were interested in whether women who use HC, and thus have comparably stable sex hormone concentrations, would also show time effects. Since the level of sex hormones over the cycle is nearly constant for the HC women (Rivera et al., 1999), no difference between cycle phase in the allocation of attention toward the most attractive and unattractive body part was expected.

Our hypotheses were partially confirmed. In line with previous studies (Haselton and Gangestad, 2006; Durante et al., 2008), naturally cycling (NC) women felt less attractive, especially for potential partners, during the late luteal phase (cycle end) relative to ovulation (mid cycle). Additionally, they gazed longer at and more frequently toward their most unattractive relative to their most attractive body part at the end of their menstrual cycle. Therefore, cycle phase seems to be associated with self-reported attractiveness and gaze pattern toward one’s own body in NC women. In particular, the most unattractive body part attracted NC women’s attention during the last days of their cycle. The observed association of menstrual cycle phase with gaze patterns toward one’s own body could play a role in the explanation of the previously reported inconsistency with regard to gaze patterns. That is, in healthy women some studies yielded evidence of a stronger focus toward unattractive parts of the own body (Janelle et al., 2003; Horndasch et al., 2012), while others observed a more balanced attention allocation for the most attractive and the most unattractive part of one’s own body (Freeman et al., 1991; Tuschen-Caffier et al., 2015). However, in all these studies menstrual cycle phase or HC were not controlled and no sex hormone levels were obtained. Consequently, future studies should control for menstrual cycle in the assessment of attentional body-related processes.

Contrary to our hypothesis, female participants were not characterized by a bias toward their most attractive body part at ovulation, although they reported higher levels of self-perceived attractiveness. A possible explanation could be that a balanced gaze distribution toward one’s own body reflects body satisfaction on a behavioral level. This would explain why only a few studies in the past found a favoring of the most attractive body part in healthy women (see the review by Rodgers and DuBois, 2016). Possibly, the self-serving bias found in these studies (e.g., Jansen et al., 2005) could have been fostered by mood, as previous studies have shown mood to significantly influence body-related attentional processing (Tuschen-Caffier et al., 2015; Svaldi et al., 2016). Alternatively, methodological issues could have contributed to these different result patterns. That is, while studies reporting a balanced gaze pattern in healthy women tested body-related attentional processing exclusively for their own body (Freeman et al., 1991; Tuschen-Caffier et al., 2015), studies that reported an attentional self-serving bias (Jansen et al., 2005) presented participants with both the self and a weight-matched other body. The latter could have instigated stronger social comparison processes, which in turn might have influenced gaze patterns concerning one’s own body.

As expected, we did not observe a significant time effect on self-assessed attractiveness and gaze patterns in our HC Group. However, for these women a general bias for the most unattractive body part was shown. Little is known about the influence of HC on body perception but studies suggest that it is associated with a higher risk of depression and a reduction in general well-being (Skovlund et al., 2016; Zethraeus et al., 2017). This could explain the more negative view of one’s own body, as depression has previously been associated with higher levels of body dissatisfaction (Marsella et al., 1981). While the investigated groups did not differ in self-reported depression and body dissatisfaction scores, they, however, differed in relationship status. Research has shown that being in a relationship influences the body perception of young women (Markey and Markey, 2006). That is, the longer women are in a relationship with their significant other, the more likely they are to incorrectly believe that their significant other wants them to look thinner. Following this finding from Markey and Markey (2006), although HC women did not report more body dissatisfaction than NC women, their appearance could have been more relevant for them because most of them were in relationships and could thus have increased their perceived pressure to look thin, thus yielding a stronger focus toward disliked body parts. Unfortunately, we did not ask participants about the duration of their relationship and whether they experience more/less perceived pressure to look thin.

Another factor that could explain why HC women showed a general bias for the most unattractive body part could be that the onset of body dissatisfaction and first intake of oral contraceptives is in early to mid adolescence (Chandra et al., 2005; Bearman et al., 2006). A relation between these two factors therefore cannot be excluded and should be addressed in future research. In comparison with the natural menstrual cycle, hormonal fluctuations in women on hormonal contraceptives are considerably smaller, particularly in users of combined oral contraceptives where ovulation is prevented. From a developmental perspective, the positive influence of ovulation on self-rated attractiveness and body-related processing could be important for the development of a positive body-related schema in puberty, where several bodily changes take place, among others an increased storage of fat cells, and body dissatisfaction increases (Moksnes and Espnes, 2012). Hence, future studies should test the effects of hormonal contraceptives on body-related attentional processing in adolescence, which is a vulnerable time for the development of many mental disorders, including eating disorders (Nagl et al., 2016). In our sample the intake time of hormonal contraceptives in the HC Group ranged from 12 to 96 months, hence some of our participants started to take hormonal contraceptives in early to late adolescence whereas some of them took it for only a short period. Due to the small sample size it was not possible to calculate separate analyses for groups with short vs. long intake times.

Notably, the detected effect sizes (partial eta squared) for the observed findings were in the small to medium range. The explained variance was between 11–41% for the main findings. Taken together, the current results are a first indication for an association of the menstrual cycle phase with body dissatisfaction. However, due to the mentioned limitations of the study, replication of the reported findings in larger samples is warranted. In addition, the design of our study was correlational. In order to test whether hormones have a causal effect on the attentional bias observed here, future studies should directly manipulate sex hormone levels for instance by administering estradiol or progesterone. Even though directional hypotheses were derived from theory and previous empirical evidence, future studies are required to replicate these findings in a larger sample with more conservatively conducted two-tailed tests in order to draw more meaningful conclusions. It cannot be ruled out that other uncontrolled state factors additionally affected participants’ perception of their own body, e.g., physically activity (Vocks et al., 2009; LePage and Crowther, 2010) or current eating habits (Mäkinen et al., 2012). These and other psychological and physical changes within the menstrual cycle could influence body perception. For example, numerous studies have shown that women feel more depressed, show more social withdrawal and are less physically active during the premenstrual phase (Sanders et al., 1983; Rubinow et al., 1984; Endicott, 1993) compared to the follicular phase. On the other hand, several studies have also found an association between these factors and body dissatisfaction (Marsella et al., 1981; Vocks et al., 2009; LePage and Crowther, 2010). Hence, given the correlational nature of our design the association and underlying mechanisms between menstrual cycle and attentional body-related processing remains largely unclear. Future studies should therefore include additional factors such as level of physical activity and social connectedness when examining the association between phase of the menstrual cycle and body image parameters.

Several other limitations need to be acknowledged. First the recruitment of women without HC within a student sample was difficult. Most likely, this was associated with a certain student lifestyle and led to a difference in relationship status between the two groups. As being in a committed relationship has previously been associated with body perception (Markey and Markey, 2006), future studies should better control for this factor. Second, our sample was restricted to young and well eductated women; therefore, it is questionable whether the results can be generalized to a wider (female) population. It has been shown that body satisfaction and perception is affected by age (Tiggemann and McCourt, 2013), with increased attention toward the most attractive body parts in older women. For example, it was reported that older adults show a preference for positive stimuli in visual attention (Isaacowitz et al., 2006). Therefore, age could be a relevant factor for the attention toward differently valued body parts. Third, the level of trait body dissatisfaction was low in our groups, which makes generalizing to at-risk or eating disordered groups difficult. Finally, we did not directly assess sex hormone levels, thus no conclusion on how estradiol and progesterone contributed to the observed effects can be drawn.

These limitations notwithstanding, the heterogeneous results that have been observed in the field of attentional bias and body image (Rodgers and DuBois, 2016; Jiang and Vartanian, 2018) together with the present results suggest that state factors such as the menstrual cycle should receive more attention in future research on automatic body-related processing. It remains unclear whether the influence of menstrual cycle phase is also relevant in the body dissatisfaction of women with eating disorders or women with high levels of body dissatisfaction. This should therefore be addressed in future studies. Should the current findings be replicated, they could have potential implications for interventions designed to enhance body image, such as optimizing the use of mirror image exposure by aligning its use with the phase of the menstrual cycle.

## Author Contributions

KK, BD, and JS contributed conception and design of the study. KK organized the database and wrote the first draft of the manuscript. KK and JS performed the statistical analysis. All authors contributed to manuscript revision, read, and approved the submitted version.

## Conflict of Interest Statement

The authors declare that the research was conducted in the absence of any commercial or financial relationships that could be construed as a potential conflict of interest.

## References

[B1] American Psychiatric Association [APA] (2013). *Diagnostic and Statistical Manual of Mental Disorders*, 5th Edn Arlington, VA: American Psychiatric Association 10.1176/appi.books.9780890425596

[B2] Asso D. (1984). The real menstrual cycle. *Br. J. Psychiatry* 145 559–559.

[B3] Bancroft J. Bäckström T. (1985). Premenstrual syndrome. *Clin. Endocrinol.* 22 313–336. 10.1111/j.1365-2265.1985.tb03244.x 4038925

[B4] Bauer A. Schneider S. Waldorf M. Braks K. Huber T. J. Adolph D. (2017). Selective visual attention towards oneself and associated state body satisfaction: an eye-tracking study in adolescents with different types of eating disorders. *J. Abnorm. Child Psychol.* 45 1647–1661. 10.1007/s10802-017-0263-z 28133705

[B5] Bearman S. K. Presnell K. Martinez E. Stice E. (2006). The skinny on body dissatisfaction: a longitudinal study of adolescent girls and boys. *J. Youth Adolesc.* 35 217–229. 10.1007/s10964-005-9010-9 16912810 PMC1540456

[B6] Beck A. T. Steer R. A. Brown G. K. (1996). *Beck Depression Inventory-II*, Vol. 78 San Antonio, TX: Psychological Corporation

[B7] Borchert J. Heinberg L. (1996). Gender schema and gender role discrepancy as correlates of body image. *J. Psychol.* 130 547–559. 10.1080/00223980.1996.9915021 8865628

[B8] Carr-Nangle R. E. Johnson W. G. Bergeron K. C. Nangle D. W. (1994). Body image changes over the menstrual cycle in normal women. *Int. J. Eat. Disord.* 16 267–273. 10.1002/1098-108X(199411)16:3<267::AID-EAT2260160307>3.0.CO;2-Y 7833960

[B9] Cash T. F. Morrow J. A. Hrabosky J. I. Perry A. A. (2004). How has body image changed? A cross-sectional investigation of college women and men from 1983 to 2001. *J. Consult. Clin. Psychol. Rev.* 72 1081–1089. 10.1037/0022-006X.72.6.1081 15612854

[B10] Chandra A. Martinez G. M. Mosher W. D. Abma J. C. Jones J. (2005). Fertility, family planning, and reproductive health of U.S. women; data from the 2002 National Survey of Family Growth. *Vital Health Stat.* 23 1–160. 16532609

[B11] Clark A. Skouteris H. Wertheim E. H. Paxton S. J. Milgrom J. (2009). The relationship between depression and body dissatisfaction across pregnancy and the postpartum: a prospective study. *J. Health Psychol.* 14 27–35. 10.1177/1359105308097940 19129334

[B12] Cooper P. J. Taylor M. J. Cooper Z. Fairbum C. G. (1987). The development and validation of the body shape questionnaire. *Int. J. Eat. Disord.* 6 485–494. 10.1002/1098-108X(198707)6:4<485::AID-EAT2260060405>3.0.CO;2-O

[B13] Durante K. M. Li N. P. Haselton M. G. (2008). Changes in women’s choice of dress across the ovulatory cycle: naturalistic and laboratory task-based evidence. *Pers. Soc. Psychol. Bull.* 34 1452–1460. 10.1177/0146167208323103 18719219

[B14] Endicott J. (1993). The menstrual cycle and mood disorders. *J. Affect. Disord.* 29 193–200. 10.1016/0165-0327(93)90033-G 8300978

[B15] Frederick D. A. Peplau L. A. Lever J. (2006). The swimsuit issue: correlates of body image in a sample of 52,677 heterosexual adults. *Body Image* 3 413–419. 10.1016/j.bodyim.2006.08.002 18089245

[B16] Freeman R. Touyz S. Sara G. Rennie C. Gordon E. Beumont P. (1991). In the eye of the beholder: processing body shape information in anorexic and bulimic patients. *Int. J. Eat. Disord.* 10 709–714. 10.1002/1098-108X(199111)10:6<709::AID-EAT2260100609>3.0.CO;2-N

[B17] Freeman R. J. Beach B. Davis R. Solyom L. (1985). The prediction of relapse in bulimia nervosa. *J. Psychiatr. Res.* 19 349–353. 10.1016/0022-3956(85)90039-1 3862835

[B18] Gao X. Deng X. Yang J. Liang S. Liu J. Chen H. (2014). Eyes on the bodies: an eye tracking study on deployment of visual attention among females with body dissatisfaction. *Eat. Behav.* 15 540–549. 10.1016/j.eatbeh.2014.08.001 25173688

[B19] Gao X. Li X. Yang X. Wang Y. Jackson T. Chen H. (2013). I can’t stop looking at them: interactive effects of body mass index and weight dissatisfaction on attention towards body shape photographs. *Body Image* 10 191–199. 10.1016/j.bodyim.2012.12.005 23352761

[B20] Garfinkel P. E. Coldbloom D. Marion R. D. Olmsted P. Garner D. M. Halmi K. A. (1992). Body dissatisfaction in bulimia nervosa: relationship to weight and shape concerns and psychological functioning. *Int. J. Eat. Disord.* 11 151–161. 10.1002/1098-108X(199203)11:2<151::AID-EAT2260110206>3.0.CO;2-Z

[B21] Grilo C. M. Masheb R. M. White M. A. (2010). Significance of overvaluation of shape/weight in binge-eating disorder: comparative study with overweight and bulimia nervosa. *Obesity* 18 499–504. 10.1038/oby.2009.280 19713949 PMC2845446

[B22] Haselton M. G. Gangestad S. W. (2006). Conditional expression of women’s desires and men’s mate guarding across the ovulatory cycle. *Horm. Behav.* 49 509–518. 10.1016/j.yhbeh.2005.10.006 16403409

[B23] Hewig J. Cooper S. Trippe R. H. Hecht H. Straube T. Miltner W. H. (2008). Drive for thinness and attention toward specific body parts in a nonclinical sample. *Psychosom. Med.* 70 729–736. 10.1097/PSY.0b013e31817e41d3 18606732

[B24] Horndasch S. Kratz O. Holczinger A. Heinrich H. Honig F. Noth E. (2012). ”Looks do matter”-visual attentional biases in adolescent girls with eating disorders viewing body images. *Psychiatry Res.* 198 321–323. 10.1016/j.psychres.2011.12.029 22417927

[B25] Isaacowitz D. M. Wadlinger H. A. Goren D. Wilson H. R. (2006). Is there an age-related positivity effect in visual attention? A comparison of two methodologies. *Emotion* 6 511–516. 10.1037/1528-3542.6.3.511 16938091

[B26] Janelle C. M. Hausenblas H. A. Ellis R. Coombes S. A. Duley A. R. (2009). The time course of attentional allocation while women high and low in body dissatisfaction view self and model physiques. *Psychol. Health* 24 351–366. 10.1080/08870440701697367 20204998

[B27] Janelle C. M. Hausenblas H. A. Fallon E. A. Gardner R. E. (2003). A visual search examination of attentional biases among individuals with high and low drive for thinness. *Eat. Weight Disord.* 8 138–144. 10.1007/BF03325003 12880191

[B28] Jansen A. Nederkoorn C. Mulkens S. (2005). Selective visual attention for ugly and beautiful body parts in eating disorders. *Behav. Res. Ther.* 43 183–196. 10.1016/j.brat.2004.01.003 15629749

[B29] Jiang M. Y. W. Vartanian L. R. (2018). A review of existing measures of attentional biases in body image and eating disorders research. *Aust. J. Psychol.* 70 3–17. 10.1111/ajpy.12161

[B30] Keel P. K. Dorer D. J. Franko D. L. Jackson S. C. Herzog D. B. (2005). Postremission predictors of relapse in women with eating disorders. *Am. J. Psychiatry* 162 2263–2268. 10.1176/appi.ajp.162.12.2263 16330589

[B31] Kimmel H. D. (1957). Three criteria for the use of one-tailed tests. *Psychol. Bull.* 54 351–353. 10.1037/h0046737 13465928

[B32] Krohne H. W. Egloff B. Kohlmann C.-W. Tausch A. (1996). Untersuchungen mit einer deutschen Version der” positive and negative affect schedule”(PANAS). *Diagnostica* 42 139–156.

[B33] Kühner C. Bürger C. Keller F. Hautzinger M. (2007). Reliabilität und Validität des revidierten beck-depressionsinventars (BDI-II). *Der Nervenarzt* 78 651–656. 10.1007/s00115-006-2098-7 16832698

[B34] LePage M. L. Crowther J. H. (2010). The effects of exercise on body satisfaction and affect. *Body Image* 7 124–130. 10.1016/j.bodyim.2009.12.002 20153709

[B35] Lewer M. Bauer A. Hartmann A. S. Vocks S. (2017). Different facets of body image disturbance in binge eating disorder: a review. *Nutrients* 9:E1294. 10.3390/nu9121294 29182531 PMC5748745

[B36] Lykins A. D. Ferris T. Graham C. A. (2014). Body region dissatisfaction predicts attention to body regions on other women. *Body Image* 11 404–408. 10.1016/j.bodyim.2014.05.003 25047004

[B37] Mäkinen M. Puukko-Viertomies L.-R. Lindberg N. Siimes M. A. Aalberg V. (2012). Body dissatisfaction and body mass in girls and boys transitioning from early to mid-adolescence: additional role of self-esteem and eating habits. *BMC Psychiatry* 12:35. 10.1186/1471-244X-12-35 22540528 PMC3370989

[B38] Markey C. N. Markey P. M. (2006). Romantic relationships and body satisfaction among young women. *J. Youth Adolesc.* 35 256–264. 10.1093/asj/sjv270 26893523

[B39] Marsella A. J. Shizuru L. Brennan J. Kameoka V. (1981). Depression and body image satisfaction. *J. Cross Cult. Psychol.* 12 360–371. 10.1177/0022022181123007

[B40] Masheb R. M. Grilo C. M. (2008). Prognostic significance of two sub-categorization methods for the treatment of binge eating disorder: negative affect and overvaluation predict, but do not moderate, specific outcomes. *Behav. Res. Ther.* 46 428–437. 10.1016/j.brat.2008.01.004 18328464 PMC3655333

[B41] Moksnes U. K. Espnes G. A. (2012). Self-esteem and emotional health in adolescents – gender and age as potential moderators. *Scand. J. Psychol.* 53 483–489. 10.1111/sjop.12021 23170865

[B42] Moos R. H. Kopell B. S. Melges F. T. Yalom I. D. Lunde D. T. Clayton R. B. (1969). Fluctuations in symptoms and moods during the menstrual cycle. *J. Psychosom. Res.* 13 37–44. 10.1016/0022-3999(69)90017-8 5813369

[B43] Nagl M. Jacobi C. Paul M. Beesdo-Baum K. Höfler M. Lieb R. (2016). Prevalence, incidence, and natural course of anorexia and bulimia nervosa among adolescents and young adults. *Eur. Child Adolesc. Psychiatry* 25 903–918. 10.1007/s00787-015-0808-z 26754944

[B44] Neumark-Sztainer D. Paxton S. J. Hannan P. J. Haines J. Story M. (2006). Does body satisfaction matter? Five-year longitudinal associations between body satisfaction and health behaviors in adolescent females and males. *J. Adolesc. Health* 39 244–251. 10.1016/j.jadohealth.2005.12.001 16857537

[B45] Pinhas L. Fok K. H. Chen A. Lam E. Schachter R. Eizenman O. (2014). Attentional biases to body shape images in adolescents with anorexia nervosa: an exploratory eye-tracking study. *Psychiatry Res.* 220 519–526. 10.1016/j.psychres.2014.08.006 25216561

[B46] Pook M. Tuschen-Caffier B. Stich N. (2002). Evaluation des Fragebogens zum Figurbewusstsein (FFB, Deutsche Version des body shape questionnaire). *Verhaltenstherapie* 12 116–124. 10.1159/000064375

[B47] Purton T. Mond J. Cicero D. Wagner A. Stefano E. Rand-Giovannetti D. (2019). Body dissatisfaction, internalized weight bias and quality of life in young men and women. *Qual. Life Res.* 10.1007/s11136-019-02140-w [Epub ahead of print]. 30783875

[B48] Racine S. E. Culbert K. M. Keel P. K. Sisk C. L. Burt S. A. Klump K. L. (2012). Differential associations between ovarian hormones and disordered eating symptoms across the menstrual cycle in women. *Int. J. Eat. Disord.* 45 333–344. 10.1002/eat.20941 21656540 PMC3170673

[B49] Rivera R. Yacobson I. Grimes D. (1999). The mechanism of action of hormonal contraceptives and intrauterine contraceptive devices. *Am. J. Obstet. Gynecol.* 181 1263–1269. 10.1016/S0002-9378(99)70120-1 10561657

[B50] Roberts S. C. Havlicek J. Flegr J. Hruskova M. Little A. C. Jones B. C. (2004). Female facial attractiveness increases during the fertile phase of the menstrual cycle. *Proc. R. Soc. Lond. B Biol. Sci.* 271(Suppl. 5), 270–272. 10.1098/rsbl.2004.0174 15503991 PMC1810066

[B51] Rodgers R. F. DuBois R. H. (2016). Cognitive biases to appearance-related stimuli in body dissatisfaction: a systematic review. *Clin. Psychol. Rev.* 46 1–11. 10.1016/j.cpr.2016.04.006 27116714

[B52] Roefs A. Jansen A. Moresi S. Willems P. van Grootel S. van der Borgh A. (2008). Looking good. BMI, attractiveness bias and visual attention. *Appetite* 51 552–555. 10.1016/j.appet.2008.04.008 18495295

[B53] Rubinow D. R. Roy-Byrne P. Hoban M. C. Gold P. W. Post R. M. (1984). Prospective assessment of menstrually related mood disorders. *Am. J. Psychiatry* 141 684–686. 10.1176/ajp.141.5.684 6538762

[B54] Ruxton G. D. Neuhäuser M. (2010). When should we use one-tailed hypothesis testing? *Methods Ecol. Evol.* 1 114–117. 10.1111/j.2041-210X.2010.00014.x

[B55] Sanders D. Warner P. Backstrom T. Bancroft J. (1983). Mood, sexuality, hormones and the menstrual cycle. I. Changes in mood and physical state: description of subjects and method. *Psychosom. Med.* 45 487–501. 10.1097/00006842-198312000-00003 6686332

[B56] Sherman B. M. Korenman S. G. (1975). Hormonal characteristics of the human menstrual cycle throughout reproductive life. *J. Clin. Investig.* 55 699–706. 10.1172/JCI107979 1120778 PMC301805

[B57] Silverthorn D. U. Ober W. C. Garrison C. W. Silverthorn A. C. Johnson B. R. (2004). *Human Physiology: An Integrated Approach*, 3rd Edn San Francisco, CA: Pearson Education.

[B58] Skovlund C. W. Mørch L. S. Kessing L. V. Lidegaard LØ (2016). Association of hormonal contraception with depression. *JAMA Psychiatry* 73 1154–1162. 10.1001/jamapsychiatry.2016.2387 27680324

[B59] Smink F. R. E. van Hoeken D. Hoek H. W. (2012). Epidemiology of eating disorders: incidence, prevalence and mortality rates. *Curr. Psychiatry Rep.* 14 406–414. 10.1007/s11920-012-0282-y 22644309 PMC3409365

[B60] Stice E. (2002). Risk and maintenance factors for eating pathology: a meta-analytic review. *Psychol. Bull.* 128 825–848. 10.1037/0033-2909.128.5.825 12206196

[B61] Stice E. Marti C. N. Durant S. (2011). Risk factors for onset of eating disorders: evidence of multiple risk pathways from an 8-year prospective study. *Behav. Res. Ther.* 49 622–627. 10.1016/j.brat.2011.06.009 21764035 PMC4007152

[B62] Stice E. Shaw H. E. (2002). Role of body dissatisfaction in the onset and maintenance of eating pathology: a synthesis of research findings. *J. Psychosom. Res.* 53 985–993. 10.1016/S0022-3999(02)00488-9 12445588

[B63] Sundström Poromaa I. Gingnell M. (2014). Menstrual cycle influence on cognitive function and emotion processing—from a reproductive perspective. *Front. Neurosci.* 8:380 10.3389/fnins.2014.00380 PMC4241821 25505380

[B64] Svaldi J. Bender C. Caffier D. Ivanova V. Mies N. Fleischhaker C. (2016). Negative mood increases selective attention to negatively valenced body parts in female adolescents with anorexia nervosa. *PLoS One* 11:e0154462. 10.1371/journal.pone.0154462 27123587 PMC4849648

[B65] Tiggemann M. McCourt A. (2013). Body appreciation in adult women: relationships with age and body satisfaction. *Body Image* 10 624–627. 10.1016/j.bodyim.2013.07.003 23954196

[B66] Tuschen-Caffier B. Bender C. Caffier D. Klenner K. Braks K. Svaldi J. (2015). Selective visual attention during mirror exposure in anorexia and bulimia nervosa. *PLoS One* 10:e0145886. 10.1371/journal.pone.0145886 26714279 PMC4700997

[B67] Vitousek K. B. Hollon S. D. (1990). The investigation of schematic content and processing in eating disorders. *Cogn. Ther. Res.* 14 191–214. 10.1007/BF01176209

[B68] Vocks S. Hechler T. Rohrig S. Legenbauer T. (2009). Effects of a physical exercise session on state body image: the influence of pre-experimental body dissatisfaction and concerns about weight and shape. *Psychol. Health Psychol.* 24 713–728. 10.1080/08870440801998988 20205022

[B69] von Wietersheim J. Kunzl F. Hoffmann H. Glaub J. Rottler E. Traue H. C. (2012). Selective attention of patients with anorexia nervosa while looking at pictures of their own body and the bodies of others: an exploratory study. *Psychosom. Med.* 74 107–113. 10.1097/PSY.0b013e31823ba787 22210238

[B70] Waadt S. Laessle R. G. Pirke K. M. (2013). *Bulimie: Ursachen und Therapie*. Berlin: Springer-Verlag.

[B71] Watson D. Clark L. A. Tellegen A. (1988). Development and validation of brief measures of positive and negative affect: the PANAS scales. *J. Pers. Soc. Psychol.* 54 1063–1070. 10.1037/0022-3514.54.6.1063 3397865

[B72] WHO (2000). *Obesity: Preventing and Managing the Global Epidemic. Report of a WHO Consultation. WHO Technical Report Series 894*. Geneva: World Health Organization. 11234459

[B73] Williamson D. A. White M. A. York-Crowe E. Stewart T. M. (2004). Cognitive-behavioral theories of eating disorders. *Behav. Modif.* 28 711–738. 10.1177/0145445503259853 15383683

[B74] Yen S. S. C. (1979). Neuroendocrine regulation of the menstrual cycle. *Hosp. Pract.* 14 83–97. 10.1080/21548331.1979.11707503 468212

[B75] Zethraeus N. Dreber A. Ranehill E. Blomberg L. Labrie F. von Schoultz B. (2017). A first-choice combined oral contraceptive influences general well-being in healthy women: a double-blind, randomized, placebo-controlled trial. *Fertil. Steril.* 107 1238–1245. 10.1016/j.fertnstert.2017.02.120 28433366

